# Health Care Seeking Behavior for Common Childhood Illnesses in Jeldu District, Oromia Regional State, Ethiopia

**DOI:** 10.1371/journal.pone.0164534

**Published:** 2016-10-14

**Authors:** Tufa Kolola, Takele Gezahegn, Mesfin Addisie

**Affiliations:** 1 Institute of Medicine and Health Sciences, Debre Berhan University, Debre Berhan, Ethiopia; 2 School of Public Health, Addis Ababa University, Addis Ababa, Ethiopia; Institute for Health & the Environment, UNITED STATES

## Abstract

**Background:**

Even though health care seeking interventions potentially reduce child mortality from easily treatable diseases, significant numbers of children die without ever reaching a health facility or due to delays in seeking care in Ethiopia. This study aimed to assess health care seeking behavior for common childhood illnesses and associated factors.

**Methods:**

A community-based cross-sectional study was conducted in Jeldu District from January to February 2011. A systematic sampling method was used for sample selection. Data were collected from 422 caregivers with under-five children who experienced diseases within six weeks before the survey. Interviewer administered structured and pre-tested questionnaire which were used to collect data. Data entry and cleaning were carried out using Epi Info version 3.5.1 and analyzed using SPSS version 16. Descriptive analysis was done to determine the magnitude of health care seeking behavior. Multivariate logistic regression analyses were performed to identify associated factors.

**Results:**

A total of 422 caregivers of under-five children were participated in the study giving an overall response rate of 97.5%. Three hundred fifteen (74.6%) children sought care from health facilities for all conditions. However, only 55.4% of them were taken to health facilities as first source treatment during their illness and prompt care was also very low (13.7%). Marital status of the caregivers (AOR = 2.84; 95%CI: 1.62–4.98), number of symptoms experienced by the child (AOR = 2.04; 95%CI: 1.24–3.36) and perceived severity of the illness (AOR = 3.20; 95%CI: 1.96–5.22) were predictors of health care seeking behavior.

**Conclusion:**

Health care seeking behavior for childhood illnesses was delayed and decision to seek care from health facilities was influenced by worsening of the illnesses. Thus, community level promotion of prompt health care seeking is essential to enhance the health care seeking behavior for child hood illnesses in the locality.

## Introduction

Globally, the total number of under-five deaths declined from 12.7 million in 1990 to 5.9 million in 2015 [1–3]. Despite these achievements and the fact that most of child deaths are preventable or treatable, many countries still have unacceptably high levels of under-five mortality [3]. Of the global under-five deaths, most deaths (98.7%) arise in developing countries [1], and approximately half (49.6%) occur in sub-Saharan Africa alone in 2015 [1, 2].A large proportion of under-five deaths were from preventable and treatable diseases like acute respiratory infections, diarrheal diseases and malaria [1–6]. Most of these lives could have been saved through affordable treatment measures like antibiotics for acute respiratory infections, oral rehydration for diarrheal diseases and the use of appropriate drugs for malaria [2, 7–9]. However, significant numbers of the children continue to die without appropriate treatment and ever reaching health facility or due to delays in seeking care in developing countries [2, 6, 9, 10].

In Ethiopia, health care seeking behavior is poor and only a small proportion of children receive appropriate treatment [11]. Nationally, only 27% of under-five children with symptom of acute respiratory infection, 24.2% with fever and 32% with diarrhea were taken to health facility during 5years preceding 2011[12]. Seeking appropriate health care for common childhood illnesses were low and delayed as well[13, 14]. Indeed, self-care and resorting to traditional healer during illnesses were commonly practiced in rural Ethiopia [11].

Despite some studies in some parts of Ethiopia about health care seeking behavior for common childhood illnesses, still there is information gap in Jeldu District for it is not researched. So, assessing health care seeking behavior for common childhood illnesses and associated factors is useful to design appropriate interventions for the improvement of child health in the area.

## Methods

### Study period and area

This study was conducted in Jeldu District from January to February 2011. Jeldu is one of the Districts in the Oromia Regional State, Ethiopia. Jeldu District has three urban and thirty eight rural kebeles. A kebele is the smallest administrative unit in Ethiopia. Gojo Town is the capital of Jeldu District; located 110 kilometres to West of Addis Ababa.

The Ethiopian health care system is a three-tier health care system that is connected to each other by a referral system. The first, second and third level in the tier are primary health care unit (PHCU), general hospital and specialized hospital respectively. A primary health care unit which is a District health care system comprise a primary hospital, health centers and health posts [15]. According to information obtained from Jeldu District Health Office, the district has 5 health centers, 14 private clinics and 38 health posts which were functional during the study period.

### Study Design and sample

A community based cross-sectional design was carried out among a sample of 422 caregivers of children aged under-five years. Sample size was determined using a single population proportion formula with a 95% confidence level, 4% margin of error and 22% estimated health care seeking behaviour in the study of similar setting by considering 5% non-response rate. The total kebeles of the district were stratified into urban and rural. Seven rural and one urban kebeles were selected by simple random sampling. The households with under-five child experienced disease in the last six weeks were identified by Health Extension Worker working in the randomly selected kebeles. Then, systematic sampling method was employed to select the sample allocated to each randomly selected kebeles.

### Data collection

Data were collected using interviewer administered structured questionnaire (S1 Appendix). Data were collected, from the caregivers of under-five children, on socio-demographic characteristics of the caregivers (age, residence, marital status, religion, ethnicity, occupation, educational status and monthly income), history of childhood illnesses and caregivers’ health care seeking behavior for common childhood illnesses. The main suggestive symptoms of common childhood illnesses; cough accompanied with difficulty of breathing for acute respiratory infections (ARI), three or more loose or watery stools per day for diarrheal diseases and fever for febrile illnesses were used to assess caregivers health care seeking behavior.

### Data quality control

The data collectors who are fluent in local language (Afan Oromo) and who know the culture of that community were recruited. Training was given for the data collectors and supervisors on how to complete the questionnaire. The questionnaire was pre-tested on similar settings which are not part of the study area and the necessary modifications were made on some items of the questionnaire.

### Data analysis

Data entry and cleaning were carried out using Epi Info version 3.5.1and analyzed by SPSS version 16. Data set underlying the findings of this paper is presented in S1 Data in SPSS format. Descriptive analysis was done to determine magnitude and distribution of health care seeking behavior for childhood illnesses. Bivariate analysis was performed to examine the association between the independent variables and health care seeking behavior. Unadjusted odds ratios with 95% confidence intervals were calculated for all variables entered into the bivariate model. The multivariate logistic regression analysis was employed to determine the independent predictors of health care seeking behavior. All variables found significantly associated with health care seeking behavior at p-value <0.05 in the bivariate analysis were entered into the multivariate model, and adjusted odds ratios with the 95% confidence intervals corresponding to variables included in a model were calculated.

### Operational definition

#### Health care seeking behaviour

Is care sought from health facilities; hospitals, health centres, private clinics or health posts for an un-well child.

#### Common childhood illnesses

In this study common childhood illnesses are acute respiratory infections (ARI), diarrheal diseases and febrile illnesses.

#### Acute respiratory infection

Is cough accompanied with difficulty of breathing for less than two weeks at any time within the six weeks before the survey.

#### Diarrhoea

If the caregivers described their sick children had three or more loose or watery stools per day at any time within the six weeks before the survey.

#### Fever

Is perceived as fever or hot body by caregivers for their sick children at any time within the six weeks before the survey.

#### Appropriate care

Care that was sought first from health facilities

#### Prompt care

Care that was sought from health facilities within 24 hours from the recognition of the illness.

#### Delay

Care that was sought from health facilities after 24 hours from the recognition of the illnesses

### Ethical consideration

The proposal of this study was ethically reviewed and approved by the Research and Ethics Committee of School of Public Health, Addis Ababa University. Ethical clearance letter was obtained from School of Public Health and submitted to Jeldu District Health Office for permission to undertake the study. Besides, verbal informed consent was obtained from all study participants prior to interview. Verbal consent was obtained because majority of our study participants cannot read and write to provide them with written informed consent. The consent procedure used was approved by ethics committee (ref SPH/0194/03) and data collection was started after each study participants confirmed their willing to participate in this study. Moreover, no personal identifiers were used on data collection questionnaire and the data obtained from the study participants were not accessed by anybody except the investigators, and were kept confidentially.

## Results

### Socio-demographic characteristics of the caregivers

A total of 422 caregivers were involved in this study giving an overall response rate of 97.5%. Three hundred forty two (81.0%) of them were currently married while about 80 (19.0%) were currently not married (never married, divorced or widowed) during the study period. Majority of the caregivers were not attended formal education followed by primary education (Table 1).

**Table 1:** Socio-demographic characteristics of the caregivers (N = 422) in Jeldu District, Oromia Regional State, January 2011.

Variables	Frequency	Percentage
Residence		
Urban	88	20.9
Rural	334	79.1
Marital status		
Currently married	342	81
Currently not married	80	19
Religion		
Orthodox	189	44.8
Protestant	209	49.5
Wakefata	24	5.7
Ethnicity		
Oromo	417	98.8
Amhara	5	1.2
Occupation		
House wife	29	6.9
Not house wife	393	93.1
Educational status		
No formal education	275	65.2
1–8 grade	131	31.0
≥9 grade	16	3.8
Households’ monthly income in Ethiopian birr		
≤600	385	91.2
>600	37	8.8

### Pattern of healthcare-seeking behavior for childhood illnesses

Of the 422 sick children, 152 (36.0%) had cough accompanied with difficulty of breathing, 259 (61.4%) had diarrhea and 295 (69.9%) had fever within six weeks preceding the survey. Nearly three-fourth (73.5%) of the children experienced two or more symptoms. Of the total children, any care was sought for 336 (79.6%); health facilities were the most common sources where care was sought from, for 315 (74.6%) sick children. Moreover, caregivers made home remedies for 233 (55.2%), purchased medicine from pharmacies for 115 (27.3%), sought religious healing for 135 (32.0%), sought traditional healer for 91 (21.6%) and sought ‘Tebel’ in local language, translated as ‘Holy water’ for 21(5.0%) children.

**Fig 1:**
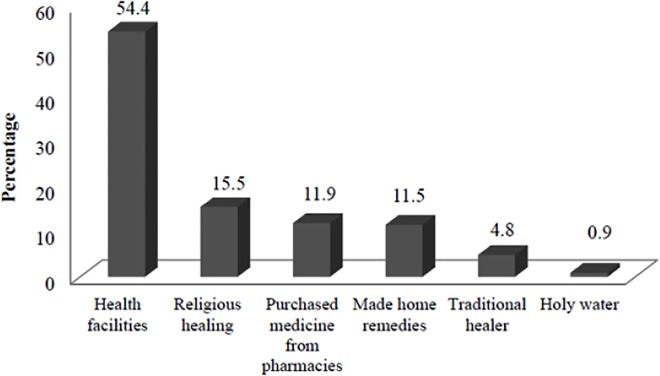
Sources where care was sought primarily for childhood illnesses (n = 336), Jeldu District, January 2011.

The most common sources where care was sought primarily were health facilities [for 186 (55.4%) children] followed by religious healing [for 52 (15.5%) children] among those who sought any care (Fig 1).

**Fig 2:**
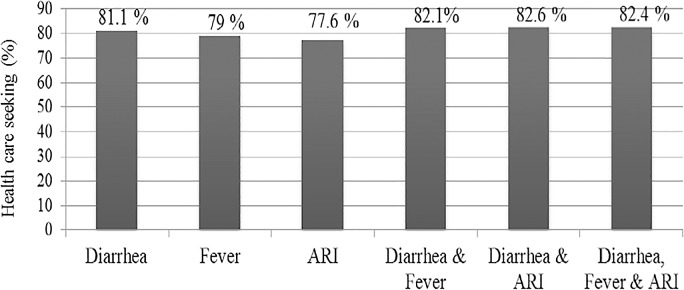
Health care seeking behaviors by conditions, Jeldu District, January 2011.

Overall, health care seeking behavior was high for the three conditions; about 81.1% of children with diarrhea, 79% of those with fever, and 77.6% of those with suggestive symptom of ARI were taken to health facilities (Fig 2).

Our finding also noted that significant proportion of children were taken to health facilities when they experienced two or more different symptoms 246 (58.3%) and when their caregivers perceived the illness as severe 192 (45.5%) (Table 2).

**Table 2:** Health care seeking behavior by number of symptom and perceived severity of the illness, Jeldu District, January 2011.

Variables	Seek care from health facilities		
	Yes (%)	No (%)	Total (%)
Number of symptom (N = 422)			
One	69(16.4)	43(10.1)	112(26.5)
Two or more	246(58.3)	64(15.2)	310(73.5)
Disease severe (N = 422)			
Yes	192(45.5)	34(8.1)	226(53.6)
No	123(29.1)	73(17.3)	196(46.4)

**Fig 3:**
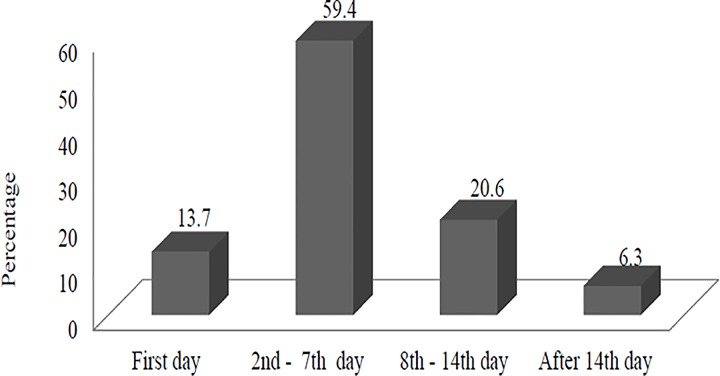
Time of health care seeking reported after recognition of the onset of the illnesses (n = 315), Jeldu District, January 2011.

Our finding shows seeking care from health facilities for sick children was delayed, only 43 (13.7%) children taken to health facilities within 24 hours after recognition of the illnesses, while for most children [272 (86.3%)], care seeking was started on the second and subsequent days (Fig 3).

Financial constraint and perception that the illness was not serious, 43(40.2%) and 29 (27.1%), were the main reasons reported for failure to seek care from health facilities respectively. The remaining 32.7% was due to caregivers’ responsibilities for all household routine which kept them busy 7 (6.5%), religious view 7 (6.5%), preference towards traditional healer 3 (2.8%), distance from health facility 2 (1.9%), treatment was expensive 1 (0.9%) and others 15 (14.0%).

### Predictors of healthcare-seeking behavior for childhood illnesses

The results of bivariate analysis showed that place of residence (OR = 0.49; 95%CI: 0.26–0.91), marital status of the caregivers (OR = 2.72; 95%CI: 1.63–4.55), number of symptoms experienced by the child (OR = 2.40; 95%CI: 1.50–3.83) and perceived severity of the disease (OR = 3.35; 95%CI: 2.10–5.34) were significantly associated with health care seeking behavior. On the other hand, caregiver’s age (OR = 0.99; 95%CI: 0.62–1.56), family size (OR = 1.00; 95%CI: 0.64–1.55), average monthly income of the households (OR = 1.84; 95%CI: 0.74–4.53), experience of child death before (OR = 0.78; 95%CI: 0.46–1.32) and sex of child (OR = 1.07; 95%CI: 0.69–1.68) had no significant association with health care seeking behavior.

In the multivariate analysis, currently married caregivers were more likely to seek care from health facilities for their sick children than those who were not married (AOR = 2.84; 95%CI: 1.62–4.98). Children who experienced two or more symptoms were more likely to receive care from health facilities as compared to those who experienced a single symptom (AOR = 2.04; 95%CI: 1.24–3.36). Caregivers were more likely to seek health care for children with diarrhea (AOR = 1.91; 95%CI: 1.20–3.06), and for those with fever (AOR = 1.79; 95%CI: 1.10–2.93). Moreover, perceived severity of the illness was associated with an increased odds of health care seeking behavior for childhood illnesses (AOR = 3.20; 95%CI: 1.96–3.06). However, being urban and rural (AOR = 0.90; 95%CI: 0.43–1.88) had no significant association with health care seeking behavior after adjusted for other variables (Table 3).

**Table 3:** Bivariate and multivariate analysis of health care seeking behavior for common childhood illnesses in Jeldu District, Oromia Regional State, January 2011.

Variables	Health care seeking		Odds Ratio (95%CI)	
	Yes	No	Unadjusted	Adjusted
Residence				
Urban	74(17.5)	14(3.3)	1	1
Rural	241(57.1)	93 (22.0)	0.49(0.26–0.91)*	0.66(0.34–1.27)
Caregivers age in years				
≤30	204(48.3)	69(16.4)	1	
>30	111(26.3)	38(9.0)	0.99(0.62–1.56)	
Marital status				
Currently married	269(63.7)	73(17.3)	2.72(1.63–4.55)*	2.84(1.62–4.98)*
Currently not married	46(10.9)	34(8.1)	1	1
Educational status of caregivers				
No formal education	206(48.8)	69(16.4)	1	
1–8 grade	95(22.5)	36(8.5)	0.88(0.55–1.42)	
≥9 grade	13(3.3)	2(0.5)	2.34(0.52–10.58)	
Family size in households				
≤ 5 persons	177(41.9)	60(14.2)	1	
>5 persons	138(32.7)	47(11.1)	1.00(0.64–1.55)	
Households’ monthly income in Ethiopian birr				
≤600	284(67.3)	101(23.9)	1	
>600	31(7.3)	6(1.4)	1.84(0.74–4.53)	
Previous child death				
Yes	82(19.4)	23(5.5)	1	
No	233(55.2)	84(19.9)	0.78(0.46–1.32)	
Sex of child				
Male	183(43.4)	64(15.2)	1	
Female	132(31.3)	43(10.3)	1.07(0.69–1.68)	
Number of symptom				
One	69(16.4)	43(10.2)	1	1
Two or more	246(58.3)	64(15.2)	2.40(1.50–3.83)*	2.04(1.24–3.36)*
Diarrhea				
Yes	210(49.8)	49(11.6)	2.34(1.52–3.70)*	1.91(1.20–3.06)*
No	105(24.9)	58(13.7)	1	1
Fever				
Yes	233(55.2)	62(14.7)	2.06(1.30–3.26)*	1.79(1.10–2.93)*
No	82(19.4)	45(10.7)	1	1
ARI				
Yes	118(41)	34(11.8)	0.94(0.54–1.65)	
No	107(37.2)	29(10.1)	1	
Disease severe				
Yes	192(45.5)	34(8.1)	3.35(2.10–5.34)*	3.20(1.96–3.06) *
No	123(29.1)	73(17.3)	1	1
Time to reach the nearest health facility on foot				
<30 minute	135(32.0)	45(10.7)	1	
30–60 minute	109(25.8)	32(7.6)	1.12(0.68–1.90)	
>60 minute	71(16.7)	30(7.1)	0.79(0.46–1.36)	

*statistically significant at p<0.05

## Discussion

This study showed that three-fourth of sick children were taken to health facilities which is higher than what have been reported previously in study conducted in Derra District, Liben District and south west of Ethiopia [14, 16, 17]. A study conducted in Malawi and Sierra Leone showed that traditional healers were frequently visited prior to others when children became ill [18, 19]. In contrary, health facilities were commonly visited prior to turn to other alternatives when children became ill in Jeldu District. Despite the better health care seeking that has been observed in our study area, appropriate health care seeking need to be improved as only 55.4% of the children taken to health facilities as first source treatment during their illness.

This study indicates that seeking care from health facilities was delayed for children presented with symptoms of acute respiratory infection, febrile illness and diarrhea. For instance, only 13.7% of the caregivers sought care from health facilities for sick children within 24 hours after recognition of the illnesses and of the total who sought care from health facilities, more than half were initiated by worsening of the illness. This finding is consistent with what has been reported by other studies in Ethiopia [13, 14], in rural Niger [20] and in Sierra Leone [19]. Similarly in Uganda, caretakers’ responses with any immediate care-giving action were delayed after they recognized the suggestive symptom of acute respiratory infection [21], while better practice was reported in a survey conducted in the Lagos Island Area, Nigeria [22]. In fact, seeking prompt and appropriate care could reduce childhood deaths that occur before the child reaches health facility or due to delays in seeking care [23–25]. The possible reasons for delayed care seeking were visiting traditional healer first, financial constraint, perceiving the illness was not serious and the expectation that illness would recover soon.

In all developing regions, child mortality and morbidity were notably higher in the lowest-income households than in wealthier households. They were also less likely to seek essential care from health facilities [6]. In this study, however, household income had no significant association with health care seeking behavior for childhood illnesses. The possible reason could be that caregivers tend to seek care from health facilities when the illnesses get worsened regardless of their monthly income. Likewise, there was no significant difference regarding distances from nearest health facility in care seeking practices from health facilities which was inconsistent with what was reported from Nigeria and Tanzania [26, 27]. This difference might be due to the availability of health posts in all selected kebeles of our study area.

The result of multivariate analysis revealed that currently married caregivers were more likely to seek care from health facilities than those who were not currently married. In Tanzania [27], caregiver’s relationship to the head of the household was predictor of health seeking behavior. Caregivers’ health care seeking behavior for childhood illnesses was influenced by number of symptoms and perceived severity of the illnesses in Jeldu District. Caregivers were more likely to seek care from health facilities when their children experienced more than one symptoms and when they perceived the illness was severe. In Kenya, perception of illness severity was strongly associated with health care seeking behavior [28, 29]. Other studies from Nigeria, Sierra Leone and Nepal also reported that multiple symptoms and perceived severity of illness were the predictors of care seeking behavior [19, 26, 30, 31].

This study was not free of limitations. The morbidity data collected were subjective in the sense that morbidity data were based on the caregivers’ perception of illness without validation by medical personnel. Moreover, this study used a recall period for six weeks and data was based on self-reported treatment seeking patterns, thus susceptible to recall bias and social desirability bias.

In conclusion, this study shows that seeking appropriate health care for childhood illnesses needs further improvement. Health care seeking behavior for childhood illnesses was delayed and decision to seek care from health facilities was influenced by worsening of the illnesses. Marital status of the caregivers, number of symptoms and caregivers perceptions about severity of illness were predictors of health care seeking behavior. Thus, community level promotion of prompt health care seeking is essential to enhance the health care seeking behavior for child hood illnesses in the locality. In addition, strengthening community-based preventive and curative health services by Health Extension Workers is vital to tackle the problem of childhood deaths from preventable and easily treatable diseases.

## Supporting Information

S1 Appendix Blank copy of the questionnaire in English. (DOCX)

S1 Data Data set underlying the findings of this paper in SPSS format. (SAV)
