# Supplementary material for: Health Care Seeking Behavior for Common Childhood Illnesses in Jeldu District, Oromia Regional State, Ethiopia
Source: PLoS One. 2016 Oct 14;11(10):e0164534. doi: 10.1371/journal.pone.0164534 (PMC5065207; doi:10.1371/journal.pone.0164534)
Supplement: S1 Appendix — (DOCX) [file pone.0164534.s001.docx]

**Information sheet and Informed consent statements**

**Information sheet**

Introduction: Hello! Sir/Madam my name is ___________and I came from_________________. We are conducting a study on health care seeking behaviour of caregivers for common childhood illnesses. The aim of this study is to collect information about health care seeking behaviour for common childhood illnesses and factors affecting it. The results of this study will be helpful for the planners to design appropriate intervention that improve the health conditions of the child in this area.

**Informed consent statements**

I am going to ask you questions about child illnesses within the previous six weeks and your health care seeking behaviour. You are randomly selected to participate in the study. Your participation in this study doesn’t involve any direct risk or benefit for you, but is very useful to improve child health in this area. Your name will not be appearing on this questionnaire, and all the information you provide me will be strictly confidential. It takes 20 minutes to finish the interview and you are not obliged to answer any question you don’t wish to answer, and you can end this interview at any time, if you wish to do so. Would you like to participate in the study?

Yes________ No_________

Interviewer, if the answer is “Yes” please let the participant sign below to certify his/her oral consent to take part voluntarily in the study. Otherwise, thank the client, conclude the conversation and file the questionnaire.

Signature_____________

Date ________________ Identification No.__________

Contact address of principal investigator: - mobile: +251921777954

Email: tufabest@gmail.com

**Questionnaires in English version**

**Section 1: Socio- demographic characteristics the care givers**

| No | Question | Coding categories |
| --- | --- | --- |
| 1 | Residence | Urban..........1  Rural............2 |
| 2 | Age of caregiver in years | __________ |
| 3 | Marital status of caregiver | Not married...1  Married..........2  Divorced........3  Widowed........4 |
| 4 | Religion | Orthodox........1  Protestant........2  Other specify__________ |
| 5 | Ethnicity | Oromo............1  Amhara...........2  Other specify__________ |
| 6 | Educational status of caregiver | No formal education......1  1 - 8 grade.....................2  ≥ 9 grade.......................3 |
| 7 | Occupation of caregiver | Farmer……...1  Government  employee.......2  Merchant........3  House wife......4  Housemaid......5  Other specify………. |
| 8 | Family size per household | <=5.............. 1  >=6.............. 2 |
| 8 | Average monthly income of the household  in Ethiopian birr | <=300.......... 1  301-600...... 2  601-900…...3  >900.............4 |
| 10 | Number of under-five children per caregiver in number | __________ |
| 11 | Have you had experience of child death before | Yes..............1  No................2 |

**Section2. Socio-demographics and health-related information of sick child**

| No |  | Coding categories | Skip to |
| --- | --- | --- | --- |
| 12 | Age of child in months | <6 ...........1  6-11........2  12-23......3  24-35......4  36-47......5  48-59......6 |  |
| 13 | Sex of the child | male .........1  female.......2 |  |
| 14 | Number of symptoms he/she has had experienced at any time in the last 6 weeks? | One................1  Two or more...2 |  |
| 15 | Has he/she had an illness with a cough at any time in the last 6 weeks? | Yes....1  No......2 | 16 |
| 16 | When he/she had an illness with a cough, did he/she have difficulty of breathing? | Yes....1  No......2 |  |
| 17 | If 25 “Yes”, for how long? | ≤2week……1  >2week……2 |  |
| 18 | Has he/she had diarrhea (three or more loose or watery stools per day) at any time in the last 6 weeks? | Yes....1  No......2 | 20 |
| 19 | Was there a blood in the stool? | Yes....1  No......2 |  |
| 20 | Has he/she had illness with a fever at any time in the last 6 weeks? (His/her body feels hot than in normal case?) | Yes....1  No......2 |  |
| 21 | Has he/she had illness with a cough and diarrhea at any time in the last 6 weeks? | Yes....1  No......2 |  |
| 22 | Has he/she had illness with a cough and fever at any time in the last 6 weeks? | Yes....1  No......2 |  |
| 23 | Has he/she had illness with diarrhea and fever at any time in the last 6 weeks? | Yes....1  No......2 |  |
| 24 | Has he/she had illness with cough with difficulty of breathing, diarrhea and fever at any time in the last 6 weeks? | Yes....1  No......2 |  |
| 25 | Was the disease severe? | Yes....1  No......2 |  |

**Section3**. **Information about health care seeking behavior of caregiver for sick child**

| 26 | According to this area sick child often taken to? | Health institution…….1  Religious area………..2  Holy water place……..3  Traditional healer…....4  I don’t know………....5 |  |
| --- | --- | --- | --- |
| 27 | Where do you prefer to seek advice or treatment if your child is sick? | Health institution…….1  Religious area………..2  Holy water place……..3  Traditional healer…….4 |  |
| 28 | Did you seek any advice or treatment for your sick child? | Yes....1  No......2 | 35 |
| 29 | Where did you seek advice or treatment?(circle all the apply) | Health facilities.............1  At home …....................2  Pharmacy.......................3  Religious area.................4  Traditional healers..........5  Holy water place................6 |  |
| 30 | Where did you seek advice or treatment first? | Health facilities.................1  Home remedies…..............2  Pharmacy...........................3  Religious area....................4  Traditional healers.............5  Holy water place.................6 |  |
| 31 | Main reason for visiting health facility? | Child’s condition worsened......1  In order not the Child’s condition get worsened ……………………..2  Other peoples’ advise..............3 |  |
| 32 | Time of health seeking after onset of the illness? | On first day..........1  Within1^st^week.......2  Wthin2^nd^week…...3  After 2^nd^week…...4 |  |
| 33 | Main reason for not visit health facility? | Lack of money.........................1  Distance from health facility....2  Illness was not serious............3  Mother busy............................4  Treatment was expensive........5  Other specify ………………… |  |

**Section 4. Information about health facility.**

34. Is there any health facility you know in this district?

Yes..........1 35

No...........2 end

35. On average how much time does it take to reach the nearest health facilities from your house on foot?

<30 minutes..........1

30-60 minutes.....2

> 1 minutes .........3

Thank you!
